# Supplementary figures and images for: The Biological Assessment of Shikonin and β,β-dimethylacrylshikonin Using a Cellular Myxofibrosarcoma Tumor Heterogeneity Model
Source: Int J Mol Sci. 2023 Nov 2;24(21):15910. doi: 10.3390/ijms242115910 (PMC10650664; doi:10.3390/ijms242115910)

supplementary data  
uncropped western blot files  
Figures 2, 4, and 6

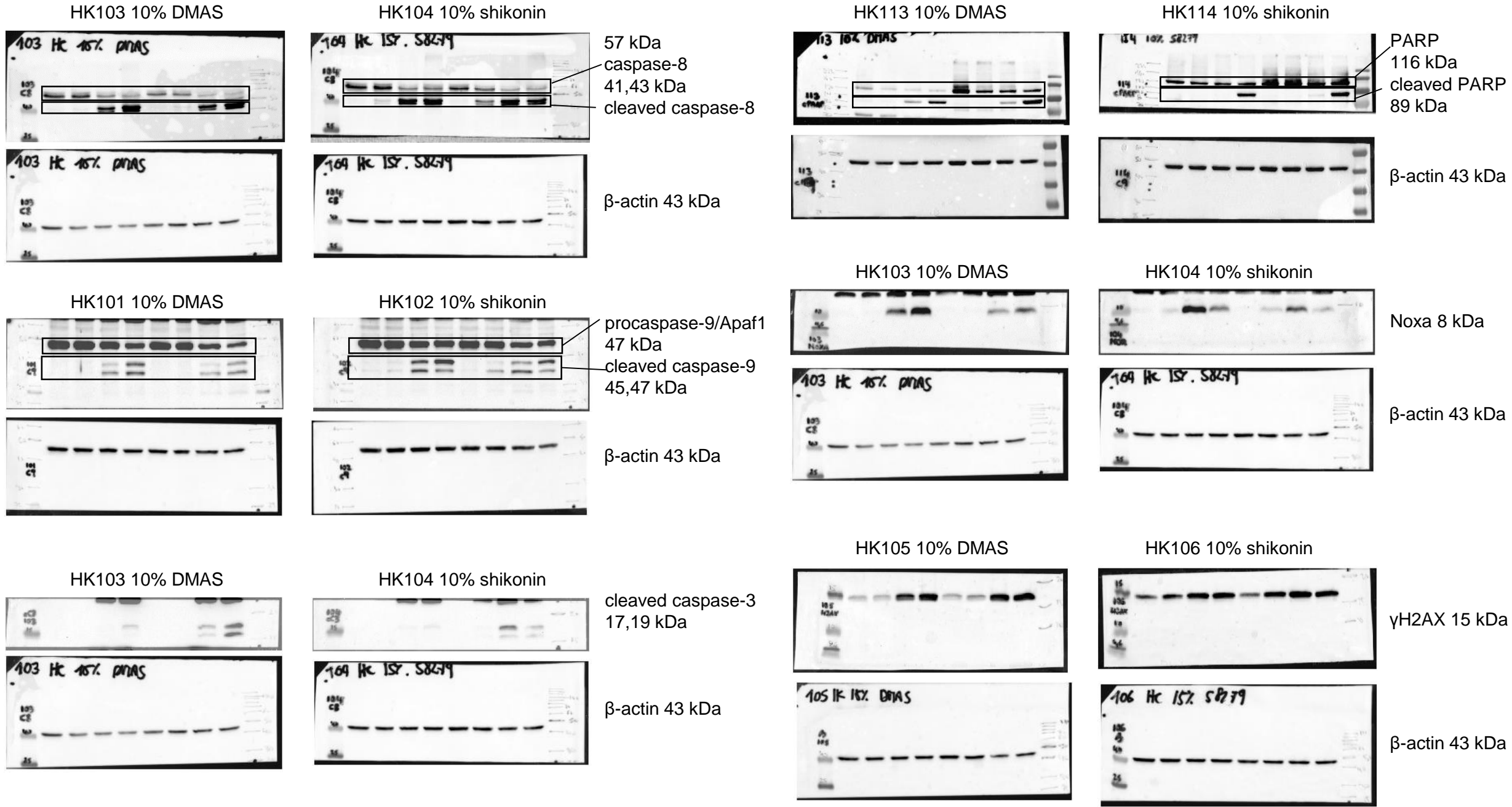

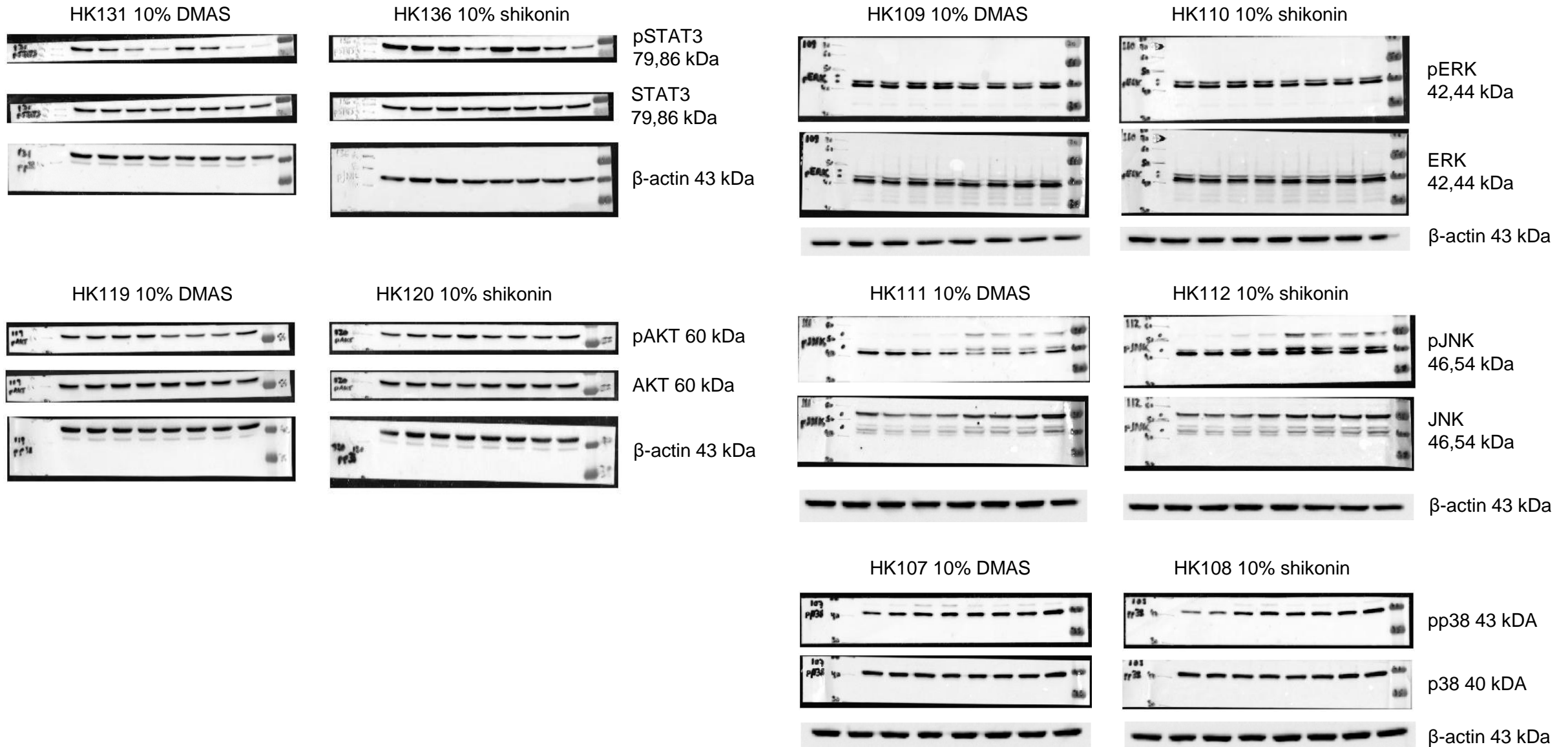

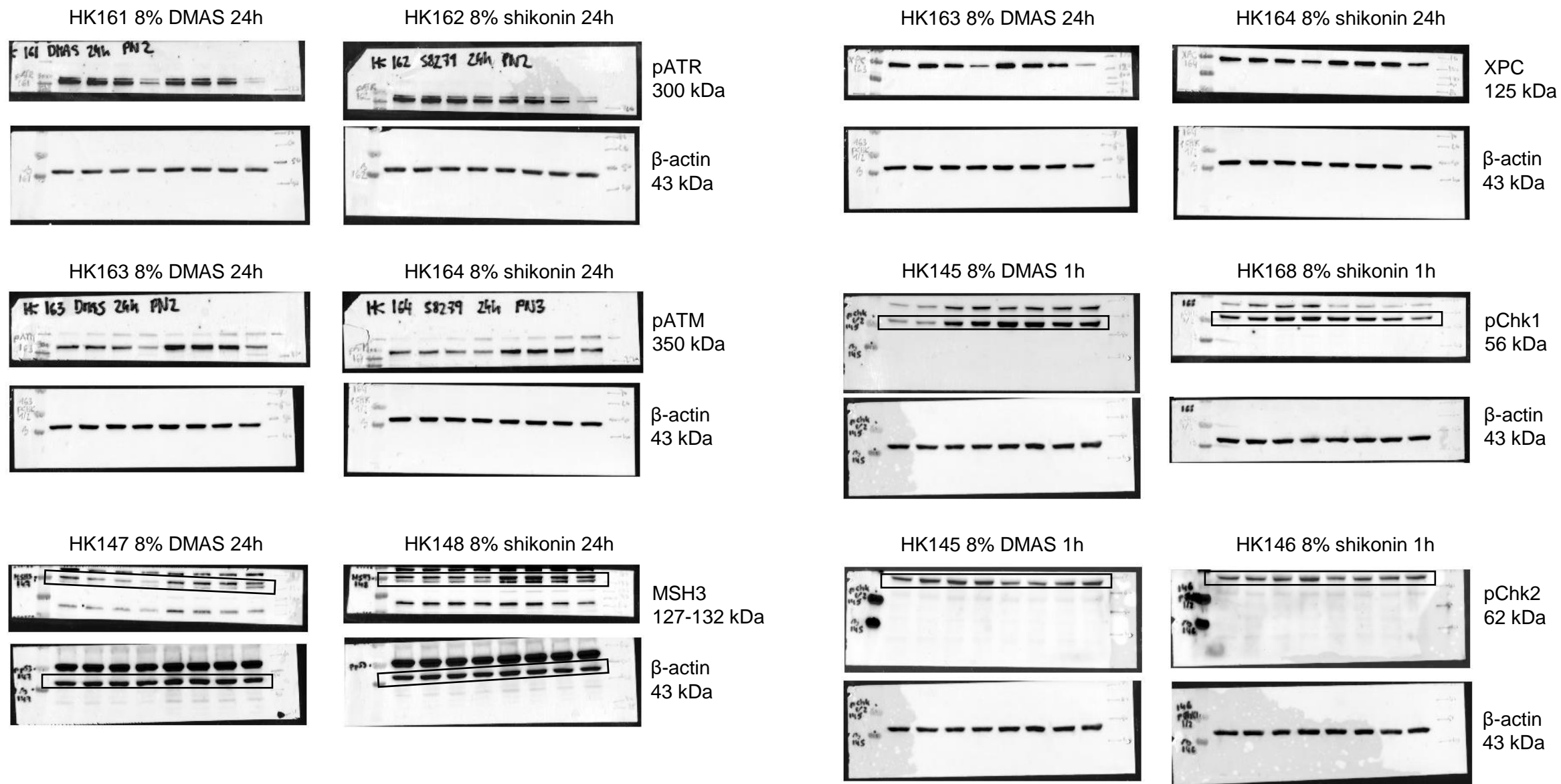

Supplement: Supplementary file 1 [file ijms-24-15910-s001.zip › ijms-2675276-supplementary.pdf]
